# Developing electronic monitor adapters for dermatologic medication containers: A methods paper

**DOI:** 10.1111/srt.13124

**Published:** 2021-11-09

**Authors:** Pooja M. Shah, Esther A. Balogh, Aaron Ross Van Gorkom, Philip J. Brown, Steven R. Feldman

**Affiliations:** ^1^ Center for Dermatology Research Wake Forest School of Medicine Winston‐Salem North Carolina USA; ^2^ Department of Biomedical Engineering Wake Forest School of Medicine Winston‐Salem North Carolina USA

**Keywords:** dermatology, electronic monitoring, medication adherence, topical medication

## 1 INTRODUCTION

Medication nonadherence is associated with increased disease severity and poor disease outcomes. Patients with skin conditions specifically may be at risk of poor disease control due to low adherence to topical treatments. Barriers to adherence include time‐consuming application, irritation, and inexact dosing.^1^

The use of the electronic monitors is the gold standard in objective evaluation of patient medication adherence. A microprocessor in the cap captures each opening and closing of the medication container and the related date and time.^2^ Electronic monitor data demonstrate significant decline in adherence to topical therapies over the treatment course for skin conditions, such as psoriasis and acne vulgaris, from 84.6 to 51% and 85 to 42%, respectively.^3^, ^4^ Physicians and patients tend to overestimate medication adherence compared to actual electronic monitor data, suggesting the importance of interventions such as patient education, frequent evaluation, motivational interviewing, and promotion of adherence.

Commercially available electronic monitors were originally designed for pill bottles and do not fit common packaging for topical drugs such as tubes and pump bottles. This creates a challenge for assessing adherence to topical ointments and creams. An adapter has been described to attach electronic monitors to tubes of medication, but that adapter is not suitable for many packages. 3D printing offers a feasible means to create adaptors for other tube sizes. Our objective was to create an adapter to affix electronic monitors to various sized containers of topical medications based on prior studies using similar adapters to assess adherence to dermatologic therapy.^5^

## 2 METHODS

**FIGURE 1:**
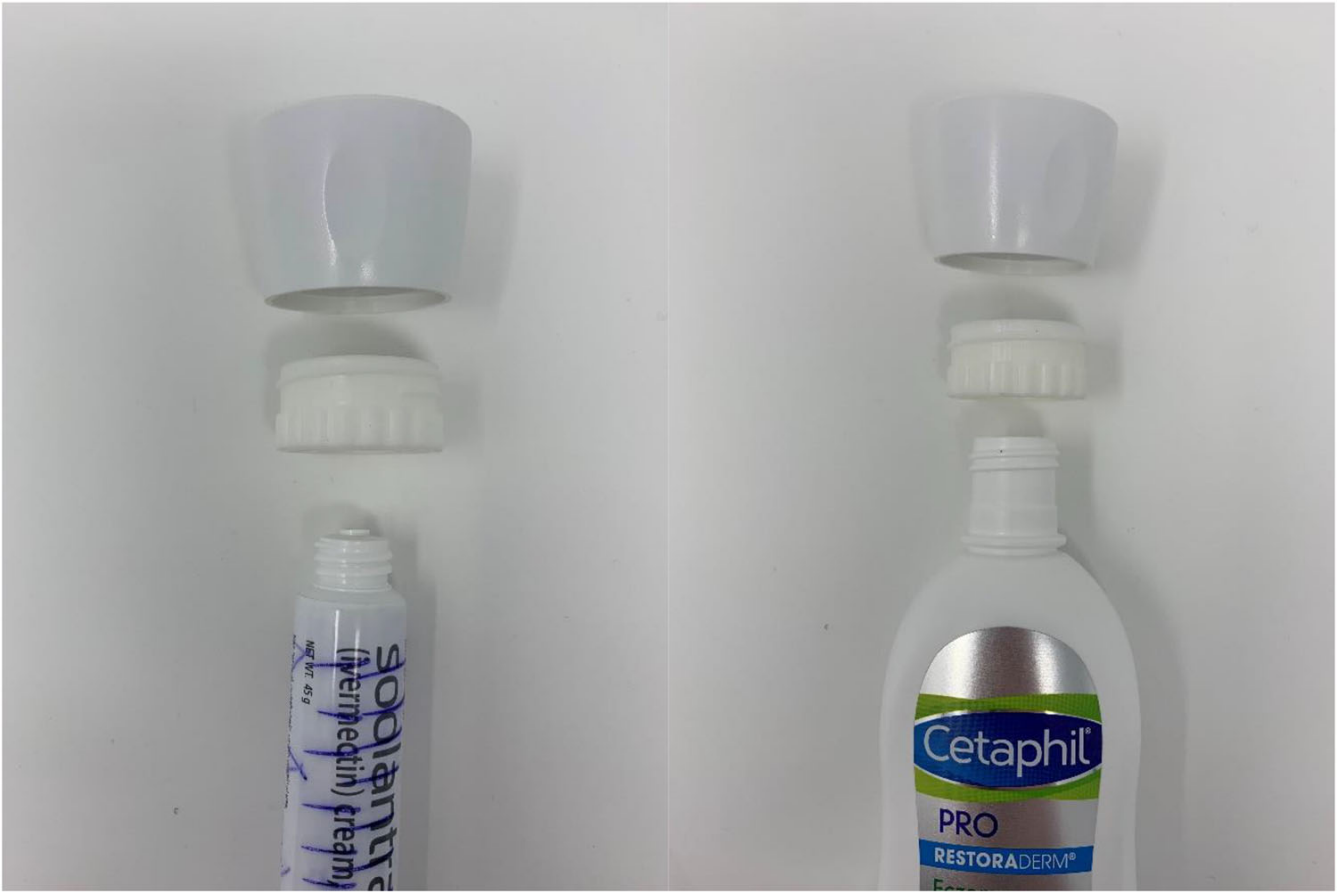
Electronic monitor adapters for 45 g and 10 fluid ounce tubes

**FIGURE 2:**
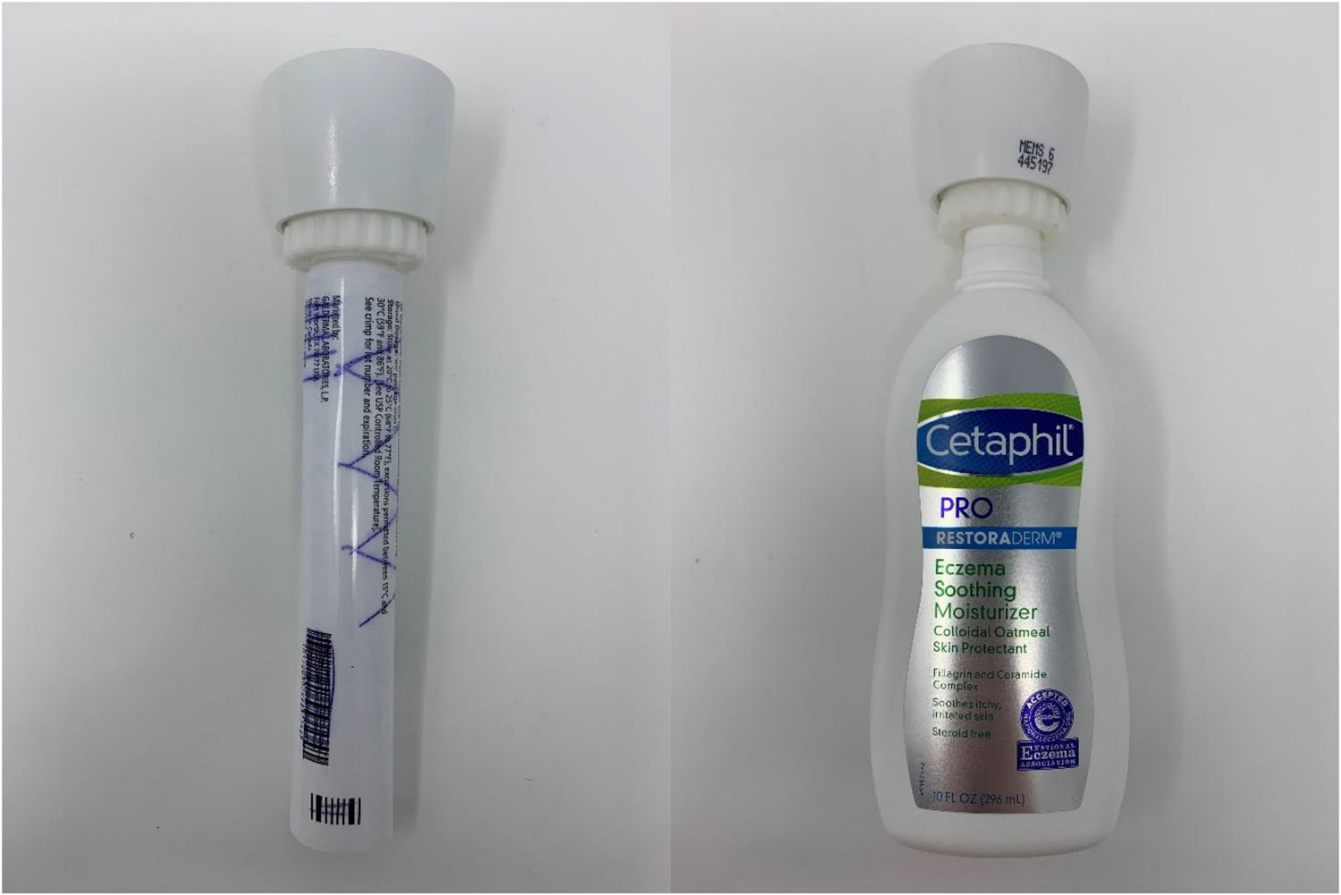
Adapters affixed to electronic monitors and 45 g and 10 fluid ounce tubes

The electronic cap adapters were reverse engineered from the two containers by taking measurements of the threads and container dimensions. The adapters were designed to work with Medication Even Monitoring System caps and were created using the Carbon M1 printer which utilizes Continuous Liquid Interface Production technology, a form of the digital light processing method of 3D printing. The material used to print the adapter was Carbon 3D's urethane methacrylate resin. Two different sizes of adapters were created to fit a 10 fl. oz. bottle of ointment and a 45 g tube of cream. The adapter was secured to each container using medical grade epoxy and threaded onto the electronic monitor cap (Figures 1 and 2). Iterative prototyping and fit testing were used to tune the 3D printed models to their specific container. The suitability of the adapters was assessed by determining if the adapters fit the electronic monitoring caps and created a water‐tight seal. Water tightness was assessed by filling each container with water and then pressurizing them by hand to ensure no leakage occurred. Accuracy testing was performed by simulating medication events and recording the percentage of openings and closings registered by the cap‐adapter system over a 1‐month trial.

## 3 RESULTS

Each custom‐sized adapter was successfully designed to meet with specific thread specifications, allowing for a water‐tight seal to be achieved between the container and the electronic monitor. The adapters created make it possible to affix the electronic monitor to topical medication containers, allowing assessment of medication adherence in upcoming studies. As the adapters are rigidly fixed to each container there is no risk of missed dosing, ensuring each studies accuracy. The 1‐month test trial yielded 100% accuracy.

## 4 DISCUSSION AND CONCLUSIONS

Medical adherence to topical therapies is relatively low compared to oral systemic medications.^6^ Particularly, since adherence tends to be overestimated by patients without monitoring, electronic monitors serve as a reliable method of assessing the role of adherence in the management of dermatologic conditions.^6^ Previous studies have shown 100% accuracy of electronic monitors confirming the opening of medication packages, consistent with this study's results.^5^, ^7^ An adapter allows the electronic monitor to fit standard cream and ointment containers, extending the use of the monitoring device to dermatologic agents. The benefit of using the adapters as opposed to patient self‐reporting of medication use is objective monitoring of patient adherence to their assigned medication protocol. Limitations of the cap‐adapter system include the monitoring only of the rate of use, not the quantity or actual application of medication; therefore, the monitors rely on the user to correctly control the dosage volume and physical application of the medication. An alternative potential design is a pump version of the electronic monitoring cap that allows for accurate dispensing of dermatological ointments in a controlled dose, thus, tracking the volume of product dispensed along with frequency of use. 3D printing is an effective method to create custom‐designed adapters which can be paired with electronic monitors to assess adherence in patients with skin conditions that require frequent and consistent application of topical medications such as psoriasis, atopic dermatitis, and acne vulgaris.^1^ These data can inform further interventions to support topical medication adherence for patients with skin conditions, who are at a disproportionate risk for nonadherence compared to patients treated with oral medication.
